# Supplementary material for: Learning curve and surgical outcome of robotic assisted colorectal surgery with ERAS program
Source: Sci Rep. 2022 Nov 29;12:20566. doi: 10.1038/s41598-022-24665-w (PMC9709162; doi:10.1038/s41598-022-24665-w)
Supplement: Supplementary file 2 — Supplementary Information 2. [file 41598_2022_24665_MOESM2_ESM.zip › RAL Raw data-Ñ[▒K/097/OP_ñw╝╨░O▒Kñσ.pdf]

正式摘要(電子簽章：000000220497137)

請選擇...reporter

臺中榮民總醫院

Operation Note

[redacted]

- 手術日期：2021/07/09
- 手術醫師：VS 蔣鋒帆，CR 陳映菁，R 陳映晴
- Anesthesia：GE
- Position：Supine
- Aseptic agents：Soap，Betadine Alcohol

Diagnosis

- Pre-operative diagnosis : Malignant tumor of A-colon
- Post-operative diagnosis : Ditto.

Surgical Indication

- tumor resection

Operation

- 1. Robotic (XI) assisted right hemicolectomy
  2. Intraoperative colonoscopy : (-)
  3. Ileo-colic artery, right colic artery and right branch of middle colic artery were all ligated via robotic endoclips.
  4. Take down from the hepatic flexure : (+)
  5. OpenGIA (80-3.8) X2 were applied to complete the side to side anastomosis
  6. Cut margin: proximal (ileum) 6 cm, (colon) 5 cm, distal 6 cm.
  7. ADHESION BARRIER FILM was placed below the fascia of the para-umbilicus wound

Finding

- 1. A 3 X 3 cm, ulcerative tumor over distal A-colon. sT4N2
  2. No other gross tumor was found.
  3. There was no visible liver metastasis or visible peritoneal seeding.

Procedure

- 1. General endotracheal anesthesia was induced and then the patient was placed in supine position.

  2. Preoperative antibiotics were given. A Foley catheter and nasogastric tube were placed.
  3. The abdomen was prepped and draped in standard sterile fashion.
  4. A small vertical incision was made below the umbilicus and a 8 mm trocar was inserted into the peritoneal cavity by open method. Peumoperitoneum was established with CO2 inflation.
  5. Laparoscopy was inserted and then other three 8mm trocars were inserted over RLQ, LUQ*2 of abdomen. Another assist-port 5mm troca was inserted on LLQ. The operative findings were mentioned as above.
  6. The small bowel was inspected and retracted to downward by grasper. The peritoneum overlying the mesentery was then scored with electrocautery and the ileocolic artery and the adjacent vein were identified, ligated with endoclips, and transected via the electrical cautery instruement of XI system
  7. omentum was freed from the transverse colon and lesser sac was entered.
  8. The right branch of middle colic artery and right colic artery were all further ligated via the electrical cautery instruement of XI system.
  9. A suspicious lymphnode was noted near middle colic artery area. It was resected for sampling.
  10. After the vessel were all under controlled, the colon was freed from its peritoneal attachments

- along the line of Toldt from the cecum to the hepatic flexure.
  11. Hemostasis was checked in the operative field. Cecum, ascending colon and hepatic flexure were mobilized and then laparoscope was removed.
  12. The midline incision was extended over right peri-umbilical area.
  13. The ascending colon was delivered into the incision and points of transection were selected proximally and distally.
  14. Side to side anastomosis was performed with two OpenGIA 80-3.8mm. Interrupted 3-0 silk Lambert sutures were used to re-inforcement at outer layer.
  15. The anastomosis was checked and found to be intact and widely patent.
  16. The small bowel and colon was put back into the abdomen. Pneumoperitoneum was established again.
  17. Possible bleeder was examed again. The orientation of the small bowel was checked thoroughly.
  18. Penrose drain was placed to pelvis.
  19. ADHESION BARRIER FILM was placed below the fascia of the central umbilicus wound
  20. The fascia was closed with interrupted sutures via Num.1 Vicryl.
  21. The skin wound was sutured with interrupted method via 3-O Vicryl, then covered with Surgicel (Gel form) and Medipore.
  22. The patient tolerated the procedure well and was brought to the POR in stable condition.
- Estimated blood loss: minimum
- 器械數量: 正確
- 紗布數量: 正確

Image

- PAINT
  - colon & rectum

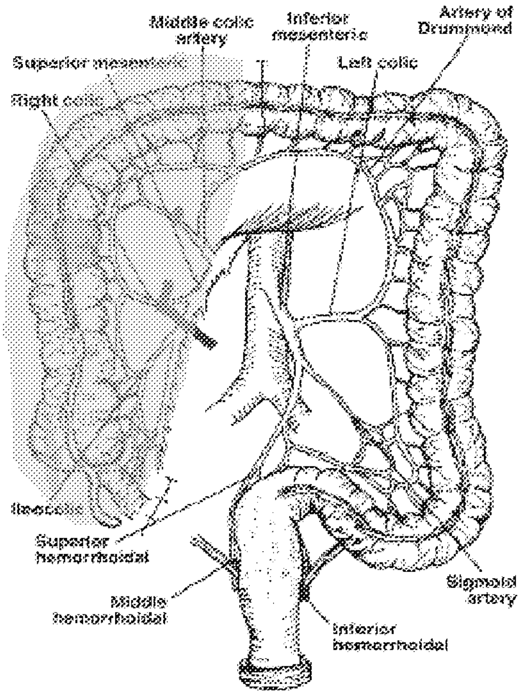

主任 2062F蔣鋒帆 / 醫師 2303K陳映菁

正式摘要(電子簽章：**000000220497137)**

請選擇...reporter

doctorRank:B
